# Supplementary material for: Environmental and Social Influences on the Behaviour of Free-Living Mandarin Ducks in Richmond Park
Source: Animals (Basel). 2022 Sep 24;12(19):2554. doi: 10.3390/ani12192554 (PMC9559490; doi:10.3390/ani12192554)
Supplement: Supplementary file 1 [file animals-12-02554-s001.zip › animals-1860073-supplementary.pdf]

Supplementary Information

Table S1: Averages + standard deviations of mandarin behaviour during different Social Periods

		Average % Time (± Standard Deviation)					
**Social Period**	**Time of Day**	**Loafing**	**Foraging**	**Preening**	**Swimming**	**Vigilance**	**Other**
Pre-Laying	08:30	39 ± 57.33	21 ± 31.64	5 ± 6.54	20 ± 20.59	7 ± 9.00	5 ± 13.70
	09:00	35 ± 50.60	18 ± 29.75	8 ± 11.82	30 ± 23.19	5 ± 8.27	4 ± 10.97
	09:30	30 ± 47.97	24 ± 32.62	12 ± 17.09	26 ± 23.92	5 ± 10.61	2 ± 5.57
	10:00	26 ± 41.22	30 ± 34.11	5 ± 7.80	23 ± 19.26	8 ± 13.77	5 ± 13.41
	13:00	33 ± 47.29	24 ± 35.23	10 ± 11.44	22 ± 17.01	5 ± 10.67	3 ± 8.49
	13:30	28 ± 49.21	23 ± 29.12	20 ± 17.04	16 ± 15.28	4 ± 8.56	4 ± 9.01
	14:00	16 ± 38.48	32 ± 38.92	14 ± 24.90	22 ± 18.43	9 ± 15.47	6 ± 12.03
	14:30	30 ± 47.42	25 ± 40.19	17 ± 25.12	18 ± 19.80	7 ± 15.00	2 ± 4.77
	16:00	37 ± 52.97	18 ± 30.67	18 ± 21.66	17 ± 20.43	9 ± 15.18	2 ± 5.88
	16:30	40 ± 58.95	14 ± 25.15	23 ± 31.50	15 ± 18.94	5 ± 11.25	2 ± 7.24
	17:00	15 ± 25.25	34 ± 42.16	8 ± 12.66	22 ± 15.18	9 ± 12.76	6 ± 17.41
	17:30	26 ± 33.37	26 ± 35.55	17 ± 23.85	13 ± 13.62	7 ± 13.06	3 ± 8.28
Laying	08:30	62 ± 37.42	6 ± 8.57	6 ± 5.99	19 ± 18.45	1 ± 1.65	0 ± 0.00
	09:00	51 ± 61.06	15 ± 24.46	12 ± 6.56	16 ± 18.04	0 ± 1.38	2 ± 4.77
	09:30	42 ± 57.29	6 ± 13.42	13 ± 17.61	35 ± 26.65	3 ± 6.92	0 ± 1.38
	10:00	36 ± 55.27	17 ± 28.85	8 ± 12.29	29 ± 23.75	2 ± 3.20	2 ± 3.50
	13:00	67 ± 63.49	8 ± 10.31	8 ± 8.14	15 ± 14.04	2 ± 3.32	2 ± 3.50
	13:30	27 ± 35.64	19 ± 25.70	4 ± 4.37	37 ± 17.32	8 ± 7.45	3 ± 4.08
	14:00	38 ± 64.17	9 ± 25.69	30 ± 37.25	10 ± 11.59	7 ± 18.03	1 ± 1.65
	14:30	50 ± 46.51	6 ± 10.28	21 ± 20.95	6 ± 6.23	6 ± 8.63	1 ± 1.75
	16:00	38 ± 16.50	17 ± 2.36	12 ± 9.43	22 ± 6.24	12 ± 2.36	0 ± 0.00
	16:30	46 ± 45.94	9 ± 19.54	21 ± 16.29	14 ± 15.70	3 ± 4.96	0 ± 2.17
	17:00	47 ± 37.14	3 ± 5.33	13 ± 13.33	21 ± 15.89	7 ± 6.71	1 ± 2.08
	17:30	46 ± 48.52	7 ± 16.38	8 ± 8.66	14 ± 14.24	8 ± 6.58	4 ± 10.43
Post-Incubation	08:30	10 ± 29.36	27 ± 33.25	5 ± 3.40	20 ± 16.53	32 ± 27.34	2 ± 4.47
	09:00	28 ± 38.02	16 ± 20.43	11 ± 12.44	19 ± 13.04	20 ± 21.79	3 ± 5.18
	09:30	64 ± 72.91	9 ± 21.47	13 ± 15.31	8 ± 16.10	5 ± 13.05	1 ± 2.86
	10:00	48 ± 72.95	4 ± 9.23	9 ± 14.67	20 ± 27.13	15 ± 23.52	2 ± 4.96
	13:00	41 ± 61.53	14 ± 20.36	13 ± 24.83	14 ± 15.27	11 ± 17.02	1 ± 1.57
	13:30	42 ± 61.03	15 ± 28.32	10 ± 16.11	16 ± 14.96	13 ± 24.68	0 ± 0.00
	14:00	21 ± 29.11	24 ± 22.47	14 ± 13.88	18 ± 16.28	16 ± 19.82	2 ± 6.08
	14:30	46 ± 70.05	13 ± 22.88	12 ± 13.27	11 ± 14.48	14 ± 20.79	1 ± 4.09
	16:00	30 ± 50.57	15 ± 24.03	15 ± 17.04	24 ± 20.16	12 ± 14.09	2 ± 5.47
	16:30	30 ± 55.96	6 ± 9.60	13 ± 14.44	24 ± 19.79	15 ± 17.46	1 ± 3.00
	17:00	16 ± 26.06	27 ± 42.32	19 ± 17.81	16 ± 13.59	17 ± 25.21	4 ± 8.31
	17:30	26 ± 55.78	23 ± 31.71	11 ± 18.25	19 ± 22.20	18 ± 19.77	2 ± 3.73
